# *Streptococcus gordonii*‐associated infective endocarditis: Case series, literature review, and genetic study

**DOI:** 10.1002/ccr3.8684

**Published:** 2024-04-05

**Authors:** Gawahir A. Ali, Andrés Pérez‐López, Clement Tsui, Khalid Shunnar, Anju Sharma, Emad B. Ibrahim, Patrick Tang, Hussam Alsoub, Wael Goravey

**Affiliations:** ^1^ Division of Infectious Diseases, Communicable Diseases Centre Hamad Medical Corporation Doha Qatar; ^2^ Department of Pathology and Laboratory Medicine Sidra Medicine Doha Qatar; ^3^ Weill Cornell Medicine in Qatar Doha Qatar; ^4^ Division of Infectious Diseases, Faculty of Medicine University of British Columbia Vancouver British Columbia Canada; ^5^ Infectious Diseases Research Laboratory National Centre for Infectious Diseases Singapore Singapore; ^6^ Lee Kong Chian School of Medicine Nanyang Technological University Singapore Singapore; ^7^ Department of cardiology Hamad Medical Corporation Doha Qatar; ^8^ Division of Microbiology, Department of Laboratory Medicine and Pathology Hamad Medical Corporation Doha Qatar; ^9^ Biomedical Research Centre Qatar University Doha Qatar

**Keywords:** biofilm, endocarditis, *Streptococcus gordonii*, WGS

## Abstract

**Key Clinical Message:**

*Streptococcus gordonii*‐associated endocarditis is a rare occurrence, raising diagnostic challenges, and is often associated with considerable morbidity. However, vigilance can prevent devastating consequences.

**Abstract:**

*Streptococcus gordonii*‐associated endocarditis is rarely reported but often associated with considerable morbidity. We describe three cases of infective endocarditis caused by *S. gordonii* during a four‐week period in 2023, and the use of whole‐genome sequencing to determine whether these isolates were genetically related. The available literature was reviewed.

## 1 INTRODUCTION

*Streptococcus gordonii* is a gram‐positive, alpha‐hemolytic streptococci that belongs to the viridans group streptococci (VGS).^1^ *S. gordonii* is part of the commensal microbiota in different body locations, including the oral cavity where it is deemed beneficial for maintaining oral health by modulating biofilm formation through competing with other VGS species implicated in dental caries such as *Streptococcus mutans*.^2^, ^3^

*S. gordonii* can also be an opportunistic pathogen and has been sporadically involved in human infections such as periodontal disease, septic arthritis, spontaneous bacterial peritonitis, and necrotizing pneumonia.^4^, ^5^, ^6^ Although infective endocarditis(IE) caused by *S. gordonii* has seldom been reported, the clinical presentation seems to be indistinguishable from that caused by other VGS.^7^

*S. gordonii* IE characteristically presents as a subacute infection, which is often complicated with subsequent valve destruction and emboli formation if not promptly recognized and treated.^8^ As in IE caused by other VGS species, penicillin remains the first‐line antibiotic treatment, which should be coupled with timely surgical intervention if indicated.^9^

Herein, we report a case series of *S. gordonii*‐associated endocarditis with different clinical courses and complications. In addition, whole‐genome sequencing (WGS) analysis was performed to determine the possible genetic relatedness among these *S. gordonii* isolates. Also, we reviewed the literature for similar cases.

## 2 METHODOLOGY

### 2.1 Cases description

#### 2.1.1 Case (1)

**FIGURE 1:**
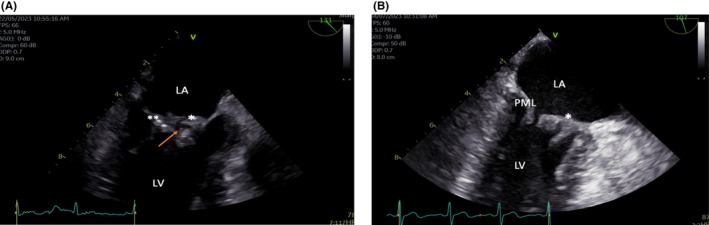
(A) TEE mid‐esophageal view with large vegetation (*) on the anterior mitral leaflet with leaflet perforation (arrow). The posterior mitral leaflet (PML) is also affected with small vegetation (**). (B) Following treatment, there is a smaller residual vegetation (*) on the anterior mitral leaflet. The PML vegetation completely resolved. LA, Left atrium; LV, Left ventricle.

A 49‐year‐old man was admitted to the hospital on May 8, 2023 with acute‐onset right‐sided weakness and global aphasia (3 h duration). Further history revealed fever and night sweats for 1 week and no other symptoms. His medical history is significant for uncontrolled diabetes mellitus and hypertension for the last 5 years. His physical examination revealed a blood pressure (BP) of 108/66 mmHg, a temperature of 38.5°C, a regular pulse rate of 92 beats per minute, a 3/6 holosystolic murmur at the apex, and right‐side hemiplegia. Laboratory tests were within normal limits except for a CRP level of 53 mg/L (0–5), and HbA1c of 9.5% (<5.7). The computed tomography of the brain revealed a large area of ischaemia in the left middle cerebral artery territory with a central core infarct. Thrombolysis was complicated by a left basal ganglia haemorrhagic transformation. A *S. gordonii* was isolated from 2 sets of blood cultures (3/4 bottles, from different venipuncture sites), which was susceptible to penicillin (MIC 0.016). A large vegetation (13 × 8 mm) with abscess formation on the mitral valve was seen on the trans‐esophageal echocardiography (TEE) with leaflet perforation, mitral valve regurgitation and a normal ejection fraction of 67% (Figure 1A). He was commenced on ceftriaxone 2 g IV every 24 h, and surgery was deemed to be a high risk given the large haemorrhagic stroke transformation. Two weeks into the therapy, the fever subsided, and a follow‐up trans‐thoracic echocardiography (TTE) showed a decrease in the size of the abscess and vegetation. Retrospectively, there was no history of dental procedure and no abnormalities on oral examination. He finished 6 weeks of ceftriaxone with a significant resolution of the vegetation and the abscess on the repeated TTE (Figure 1B).

#### 2.1.2 Case (2)

**FIGURE 2:**
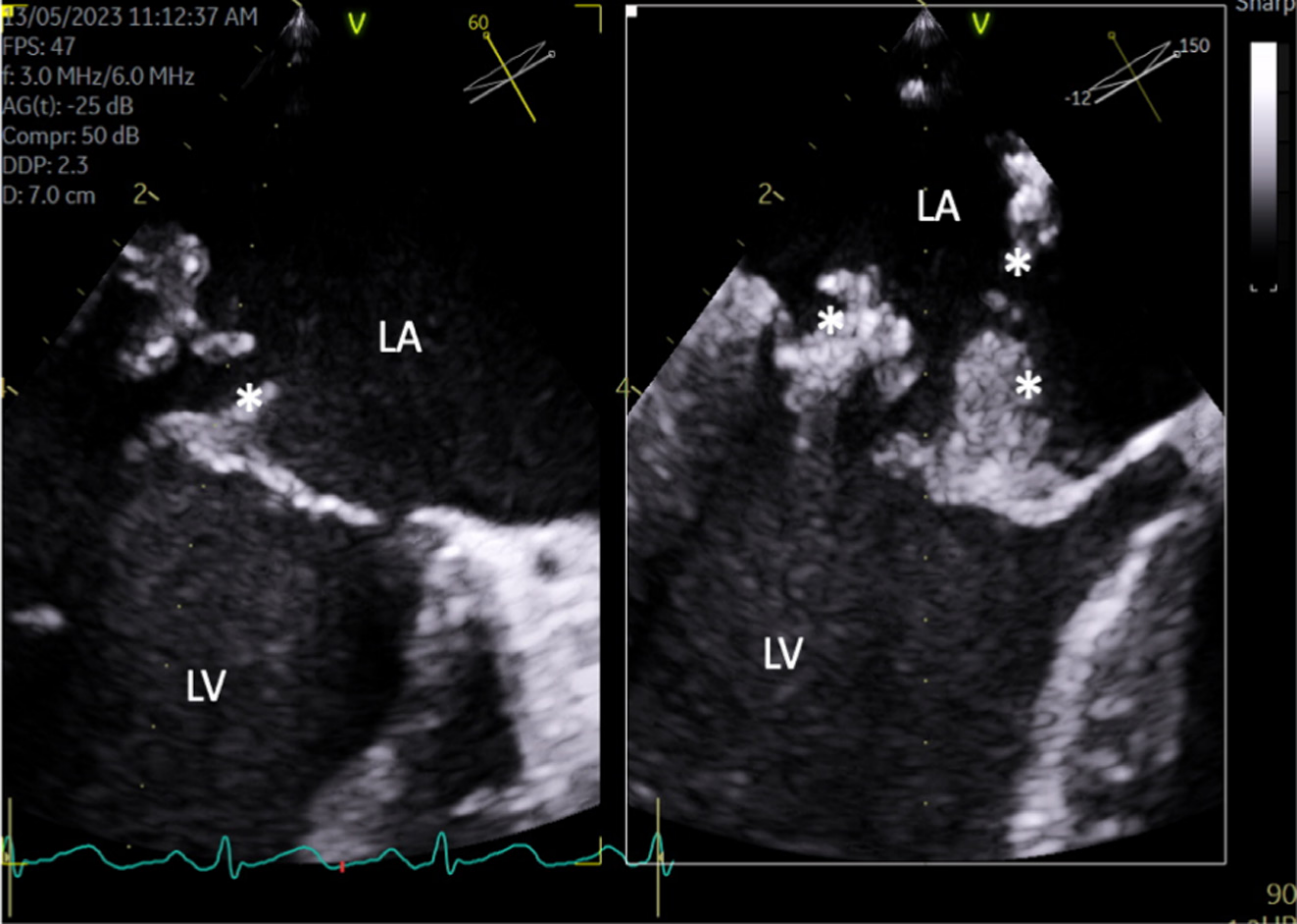
TEE mid‐esophageal views. Large, highly mobile vegetations (*) on both mitral leaflets with perforation of the posterior leaflet.

A 52‐year‐old man with no significant past medical history presented on May 11, 2023 with fever and fatigue for 8 weeks. His physical examination revealed a BP of 95/51 mmHg, a pulse rate of 58 beats per minute, and a fever (39.2°C) with a pan‐systolic murmur at the apex. His blood cultures (One set) revealed a penicillin‐susceptible *S. gordonii* (MIC 0.016), and a TEE showed vegetation on the mitral valve (3 cm), causing leaflet perforation and abscess formation with ejection fraction of 59% (Figure 2). He was treated with a 4‐week course of ceftriaxone 2 g IV every 24 h and underwent mitral valve replacement, and ceftriaxone was continued for 4 weeks. There was no history of dental procedures identified, and the patient recovered fully and was discharged home. He remained asymptomatic at the 6‐week follow‐up appointment.

#### 2.1.3 Case (3)

**FIGURE 3:**
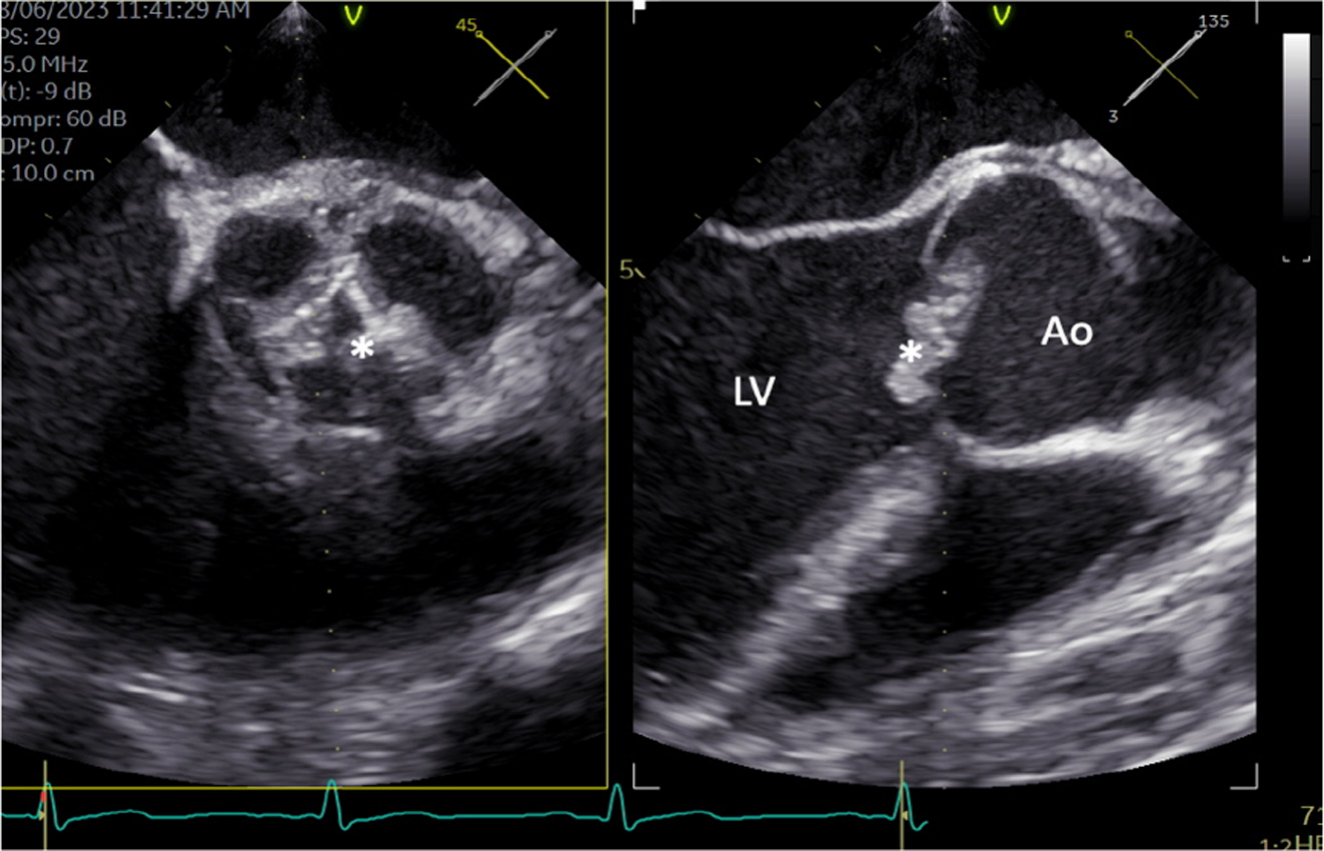
The aortic valve is bicuspid. A 10 × 3 mm vegetation is seen (*) with possible nonruptured valvular abscess. Ao, Aorta; LV, Left ventricle.

A 19‐year‐old man presented on June 5, 2023 with fever and unintentional weight loss associated with night sweats for 3 months. He had been on iron replacement therapy for the last 4 months due to his iron deficiency anemia. His physical examination revealed a BP of 96/56 mmHg, a pulse rate of 73 beats per minute, a fever of 38.1°C and hepatosplenomegaly. The cardiac examination revealed a faint systolic murmur in the aortic area. Two blood culture sets (4/4 bottles) grew a penicillin‐susceptible *S. gordonii* (MIC 0.094). A TTE revealed no evidence of vegetations and normal ejection fraction; however, a TEE showed severe aortic valve regurgitation with vegetation (10 × 3 mm) and intra‐valvular non‐ruptured abscess formation (Figure 3). Urgent aortic valve replacement was performed successfully, and he recovered fully after completing 4 weeks of treatment with ceftriaxone (2 g IV every 24 h). He traveled back to his country and was lost to follow‐up.

### 2.2 Microbiological identification and antimicrobial susceptibility testing

Microbiological identification and antimicrobial susceptibility tests (AST) were performed using matrix assisted laser desorption ionization‐time of flight mass spectrometry (MALDI‐TOF MS), Bruker Daltonics MALDI Biotyper (Billerica, MA, USA) and BD Phoenix TM system using the NMIC/ID‐94 panel according to manufacturer's instructions.

The isolates were identified as *S*. gordonii (log scores of 2.34, 2.16, and 2.14 respectively). The antibiotic susceptibility was performed as recommended by CLSI and all three isolates were susceptible to penicillin (MIC ≤0.12 μg/mL).

### 2.3 Whole‐genome sequencing (WGS) methods and interpretation

Genomic DNA was extracted using Qiagen extraction kit (Qiagen, Hilden, Germany), and quantified using Qubit 2 fluorometer (ThermoFisher, MA, USA). DNA libraries were constructed using Nextera XT library preparation kit (Illumina Inc. USA) and sequenced on the in‐house Illumina MiSeq platform with 300 bp paired‐end (Reagent kit V3). Raw reads were assessed by Fastqc (http://www.bioinformatics.babraham.ac.uk/projects/fastqc/) and quality trimmed by Trim Galore (http://www.bioinformatics.babraham.ac.uk/projects/trim_galore/) to eliminate low‐quality sequences. Trimmed reads were assembled using SPAdes v.3.9.0.^10^ Genome assemblies were assessed using QUAST v5.0.2.^11^ Genes encoding antimicrobial resistance, and the plasmids were predicted based on the ResFinder v3.2 and Plasmidfinder databases,^12^, ^13^ implemented in abricate v0.9.8 (https://github.com/tseemann/abricate). The genetic relationship between the isolates was inferred using Parsnp v1.7^14^ and compare using core genome SNPs.

**FIGURE 4:**
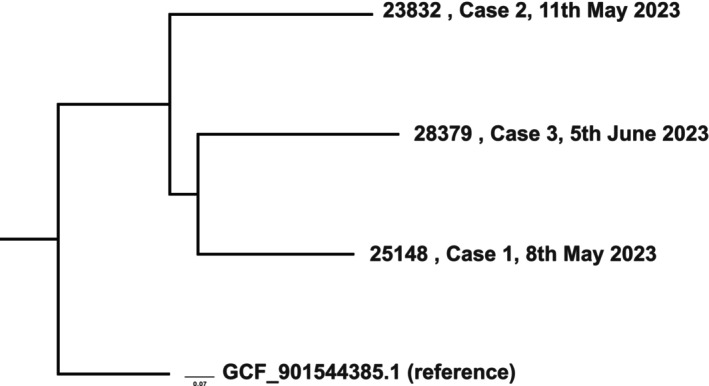
Phylogenetic tree analysis of the three *S. gordonii* isolates.

The genome size of *S. gordonii* is around 2.2 Mb, and genomic analysis were performed to determine the relationship between these 3 isolates. The core genome SNPs revealed that these 3 isolates were not similar but with >60,000 SNPs differences among them despite the occurrence within a month from two hospitals (Figure 4). Both 25,148 and 28,379 carried *tet*(*M*) that confers resistance to tetracycline and doxycycline. The genome of 28,379 also carries *msr*(*D*) and *mef*(*A*) that are responsible for resistance towards azithromycin, erythromycin, and telithromycin. The three isolates shared similar pathogenicity genes profile, including cell surface hydrophobicity proteins such as *cshA* and *cshB*, the adherence and the colonization genes including *pavA*, *srtA*, *slrA*, *plr/gapA*, *gtfG* and *lmb*, evasion from the immune system, invasion host‐tissues (*eno*), iron acquisition and uptake (*piuA*), the enzymes involved in the pathogenicity (*cppA*, *htrA/degP*, *tig/ropA*). In addition, cytolysin toxin encoded by *cylA* was detected in all 3 isolates.

## 3 DISCUSSION

Streptococci are the second most common cause of infective endocarditis after staphylococci. The viridians streptococci, which comprise several species categorized into 5 groups, is responsible for about 30% of all streptococcal‐related endocarditis.^15^ However, *S. gordonii*, which is a member of the *S. sanguinis* group, has rarely been reported to cause IE.^16^ Intriguingly, significant numbers of cases of endocarditis caused by *S. gordonii* have been reported in one study; whether this is due to species differences in that specific geographical setting or a genuine increase prevalence of *S. gordonii* endocarditis requires more investigation.^16^ Usually, oral trauma, poor dental hygiene, mucositis, and recent dental procedures are risk factors for *S. gordonii*‐associated endocarditis; however, they are rarely identified in the reported cases, as in our patients.^7^, ^17^

*S. gordonii* possesses several virulence factors facilitating the development of endocarditis. The ability to form biofilms, which is mediated by proteins PadA and Hsa, allows *S. gordonii* to efficiently bind to endothelial cells of heart valves and platelets, developing complex biofilms containing bacterium‐platelet‐fibrin complexes.^18^ Another potential virulence factor is a serine‐rich glycoprotein, GspB, in the cell wall of *S. gordonii*, which leads to further platelet aggregation.^19^ Moreover, *S. gordonii* induces a nuclear factor‐kappa B signaling pathway in the valve interstitial cells and the TLR2 signaling pathway through nitric oxide production, leading to excessive inflammatory conditions and further facilitating biofilm formation.^20^, ^21^ For these reasons, antimicrobials might fail to penetrate this unique multilayer biofilm, leading to delaying source control.^8^ This particular ability of *S. gordonii* to form biofilms might explain why 2 out of 3 of our patients had large vegetations and valve complications that required surgery.^16^ Additionally, the formation of this distinct multilayer biofilm containing platelet aggregates might explain the increased rate of embolic events associated with *S. gordonii* IE^22^; However, Chamat‐Hedemand et al reported a low prevalence of embolization in patients with *S. gordonii* IE.^8^ Noticeably, ischemic stroke, secondary to embolic events, is the presenting symptom in 20% of all streptococci endocarditis, as observed in the first case.^23^ Chamat‐Hedemand et al, reported that the most common presenting symptoms for VGS‐related IE were fever and heart murmur at 86% and 81%, respectively.^8^ They also found that, the aortic valve was infected in about half, the mitral valve in 36%, and both aortic and mitral valves in 17% of all VGS‐related IE.^8^ Despite all these characteristics, it is not possible to differentiate between IE caused by *S. gordonii* from that caused by other members of the VGS based on clinical, laboratory, and echocardiographic features. It is noteworthy that, when *S. gordonii* bacteremia is complicated by IE, it is more likely associated with multivalve involvement and younger age when compared with other VGS‐related IE.^16^ Almost all our patients presented with fever and murmurs, with involvement of the mitral valves in two out of the three cases.

It is also worth noting that matrix‐assisted laser desorption‐ionization time‐of‐flight mass spectrometry (MALDI‐TOF MS) has demonstrated to accurately identify viridans streptococci within the sanguinis group to the species level unlike the S. mitis group.^24^ The in‐hospital mortality for VGS‐related IE ranges from 10% to 29%, depending on patient characteristics and complications.^8^

The treatment of choice for highly penicillin‐susceptible *S. gordonii* native valve endocarditis is a 4‐week regimen of parenteral penicillin or ceftriaxone. Gentamicin should be added for the first 2 weeks of therapy if isolates with intermediate susceptibility to penicillin are detected.^9^ Timely surgical intervention is crucial to improve prognosis and embolic events.^25^

Interestingly, novel therapeutic strategies that target major virulence factors, virulence‐mediated pathways, and biofilm formation of *S. gordonii* could be a promising alternative or add‐on to conventional antimicrobial therapy; however, further research is warranted to better understand the pathogenesis, molecular characteristics, and practicality of using these novel therapies.^26^

Our patients were young, had native heart valves, no history of dental disease, and presented during four‐week period. Therefore, we used WGS to investigate this cluster of cases.

The small sample size limits the study generalizability and the accuracy of identifying single reference genome for SNPs when analyzing WGS data for epidemiological purposes, limiting standardization of the study.

We searched the PubMed, Embase, and Cochrane Library databases in November 2023 for similar cases. The search terms included “Streptococcus gordonii,” “bacteremia,” and “endocarditis,”. We excluded infections caused by *S. gordonii* other than endocarditis. The search was restricted to articles written in English and yielded a total of 25 cases of *S. gordonii* ‐related endocarditis (Table 1). Cases ranged between 11 and 83 years of age and were predominantly males. Four patients had recent dental procedures,^7^, ^22^, ^27^, ^28^ while only one reported a history of recently treated tonsillitis.^29^ Of all patients reviewed, only three had recent valve replacement/repair or abnormal valves as a risk factor for endocarditis.^7^, ^30^, ^31^

**TABLE 1:** Summary of previously reported cases of *S. gordonii* endocarditis.

	Case	Age/Gender	Risk factors	Symptoms/Duration in weeks	Valves involve/Vegetation size	Valve complications	Pen MIC	Embolic phenomenon	Treatment/Duration	Valve surgery	Outcome
1	Dadon et al., 1998^7^	23/F	NA	Fever/NA	MV	–	–	–	Gentamicin/2 weeks Penicillin/4 weeks	No	A live
2	Dadon et al., 2006^7^	37/M	NA	Exertional dyspnea/NA	AV	–	–	–	Not reported	Yes	Die
3	Dadon et al., 2006^7^	45/M	NA	Fever/NA	MV	–	–	–	Gentamicin/1 week Penicillin/6 weeks	Yes	A live
4	Dadon et al., 2007^7^	75/M	NA	Fever/NA	MV + AV	–	–	–	Penicillin/6 weeks	No	A live
5	Dadon et al., 2013^7^	83/F	NA	Fever/NA	MV	–	–	–	Ceftriaxone/6 weeks	No	A live
6	Dadon et al., 2014^7^	78/M	NA	FUO/NA	MV + AV	–	–	–	Penicillin/6 weeks	Yes	A live
7	Dadon et al., 2014^7^	71/M	NA	Fever/NA	–	–	–	–	Penicillin/6 weeks	No	A live
8	Dadon et al., 2015^7^	31/M	NA	Fever/NA	MV	–	–	–	Penicillin/4 weeks	Yes	A live
9	Dadon et al., 2016^7^	82/M	NA	Back pain/NA	MV	–	–	Discitis	Penicillin/12 weeks	No	A live
10	Dadon et al., 2016^7^	63/M	None	Back pain/NA	AV	–	–	Discitis	IV penicillin/8 weeks + oral amoxicillin/4 weeks	No	A live
11	Ikeda et al., 2016^34^	45/M	Aorto‐right atrial fistula following rupture of a sinus of Valsalva aneurysm	Fever, general fatigue, and weight loss/12 weeks	String‐shaped vegetation was attached to the tip of the windsock deformity		–	None	Gentamicin/2 weeks Ceftriaxone/4 weeks	Yes	A live
12	Dadon et al., 2017^7^	63/M	Recent periodontal debridement	Low back pain/2 week	AV/13 mm	None	–	Discitis	Gentamicin/2 weeks Penicillin/6 weeks	No	A live
13	Dadon et al., 2017^7^	71/M	NA	General deterioration/NA	AV	–	–	None	Penicillin/6 weeks	Yes	A live
14	Dadon et al., 2017^7^	82/M	Biological aortic valve replacement, mitral valve repair	Low back pain/2 week	MV/14 mm	None	–	Discitis ‐Lumbosacral epidural abscess with cord compression)	Penicillin/8 weeks	No	A live
15	Pairan et al., 2018^17^	38/F	None	Fever associated with vomiting, loss of appetite and weight loss /4 weeks	MV/0.9 cm	Severe MR	–	Brain	Penicillin/6 weeks	No	A live
16	Komorovsky et al., 2019^29^	11/F	Recent tonsillitis/pharyngitis	Fever, weakness and pain in left ankle joint/NA	MV	MVR and prolapse	‐	None	Not reported	Yes	A live
17	Mosailova et al., 2019^27^	31/M	Recent dental procedure to drain abscess	Bilateral lower extremity edema/2 days	MV	Severe MVR	–	None	Ceftriaxone/6 weeks	Yes	A live
18	Wang et al., 2020^33^	39/F	None	Fever and headache for the/24 weeks	MV	None	–	Brain	Vancomycin/2 weeks	No	Die
19	Hristakos et al., 2021^30^	64/M	Chronic severe mitral prolapse and mitral regurgitation (MR) status post MitraClip placement	Chills and rigors/1 day	MV/10 × 15 mm	Severe MVR and AVR	–	None	Vancomycin/Not reported	Yes	–
20	Arbune et al., 2021^35^	23/F	Frequent streptococcal angina	Fever, asthenia, intense sweating, myalgias, arthralgia, frontotemporal headache, pain in the left lower extremity, and weight loss/6 weeks	AV/0.5 cm	None	–	None	Ceftriaxone/6 weeks	No	A live
21	Chang et al., 2021^32^	28/M	None	Right‐sided body weakness/8 weeks generalized, colicky abdominal pain and fever/1 week	AV/1.6 cm × 0.8 cm + MV/0.5 cm × 0.8 cm	Severe AVR, moderate MVR	0.016	Multiple sites, including the brain, mesentery, kidney and spleen.	Gentamicin/Not reported benzylpenicillin/6 weeks	No	A live
22	Yan et al., 2022^36^	30/F	None	Recurrent fever and left lumbosacral pain/12 weeks	MV	None	‐	None	Ceftriaxone/4 weeks Levofloxacin lactate/2 weeks	No	A live
23	Hussin et al., 2022^28^	20/M	Dental extraction 1‐month	Non‐specific chest pain/1 week	MV/10.5 mm + AV/0.9 mm	Severe MVR + AVR	‐	Multiple sites, brain, spleen, and lower limb	Ceftriaxone and crystalline penicillin/6 weeks	Yes	A live
24	Chawla et al., 2023^22^	27/M	Self‐extracted his molar 2 months	Severe headache and total loss of vision in the left eye/4 days	MV	MVR	‐	Brain‐CRAO	Ceftriaxone/6 weeks	No	A live
25	Qu et al., 2023^31^	61/M	Congenital heart valve lesions	Fever, chills and malaise /16 weeks	AV	Aortic valve redundancy with perforation	‐	None	Not reported/4 weeks	Yes	A live
	Our case, 2023	49/Male	None	Acute right sided weakness	MV	Perforation and early abscess formation	0.016	Brain	Ceftriaxone/6 weeks		A live
	Our case, 2023	52/Male	None	Generalized body pain and weakness, fever and loss of appetite/8 weeks	MV	Destruction, perforation and ruptured abscess	0.016	None	Ceftriaxone/4 weeks	Yes	A live
	Our case, 2023	19/Male	None	Generalized fatigue, unintentional weight loss, fever associated with night sweats, left upper quadrant dragging pain, and exertional dyspnea/12 weeks	AV	AVR and abscess	0.094	None	Ceftriaxone/6 weeks	Yes	A live

Abbreviations: AV, aortic valve; AVR, aortic valve regurgitation; IE, infective endocarditis; MV, mitral valve; MVR, mitral valve regurgitation; NA, Not available.

Of the 25 cases reviewed, 64% reported a history of fever between 1 day and up to 24 weeks prior to presentation; however, in the remainder, the duration was not mentioned.^7^, ^29^

Mitral valves were most commonly involved (52%), and in almost one‐fifth of the cases, both the aortic and mitral valves were involved.^7^, ^28^, ^32^ Almost a third of the patients developed valve complications in the form of dysfunction or perforation, although the data were not available in almost half of the cases.^17^, ^22^, ^27^, ^28^, ^29^, ^30^, ^31^, ^32^

Septic emboli were reported in 36% (9 cases), of which 20% (5 cases) were settled in the brain, and 16% (4 cases) were evident in the spine, causing discitis.^7^

The duration of therapy ranged from 4 to 12 weeks, depending on the complication, although data were not always available. Only two deaths were identified in our review,^7^, ^33^ though 44% of the cases required valve surgery, either replacement or repair (Table 1).

## 4 CONCLUSION

In summary, IE caused by *S. gordonii* is unusual and often associated with embolic events, and valve complications that require surgery, which is likely related to the unique ability of this species to attach and form biofilms along with the presence of distinct virulence factors. Therefore, timely recognition, early antimicrobial therapy, and a multidisciplinary approach involving infectious disease physicians, cardiologists, and cardiac surgeons are crucial to avoid valve destruction and detrimental consequences.

## AUTHOR CONTRIBUTIONS

**Gawahir A. Ali:** Conceptualization; data curation; formal analysis; funding acquisition; investigation; methodology; project administration; resources; validation; visualization; writing – original draft; writing – review and editing. **Andrés Pérez‐López:** Data curation; methodology; resources; validation; writing – review and editing. **Clement Tsui:** Data curation; formal analysis; methodology; resources; validation; writing – review and editing. **Khalid Shunnar:** Data curation; investigation; validation; writing – review and editing. **Anju Sharma:** Data curation; formal analysis; validation. **Emad B. Ibrahim:** Data curation; funding acquisition; investigation; resources. **Patrick Tang:** Data curation; funding acquisition; methodology; resources; validation; writing – review and editing. **Hussam Alsoub:** Conceptualization; data curation; investigation; methodology; supervision; validation; writing – review and editing. **Wael Goravey:** Conceptualization; data curation; formal analysis; investigation; methodology; supervision; validation; visualization; writing – original draft; writing – review and editing.

## FUNDING INFORMATION

No funding was received toward the publication.

## CONFLICT OF INTEREST STATEMENT

The authors declare that they have no competing interests.

## ETHICS STATEMENT

Ethics approval and permission was obtained to publish the cases report from the institutional review board which is in line with international standards.

## CONSENT

Written informed consent was obtained from the patient to publish this report in accordance with the journal's patient consent policy.

## Data Availability

The raw sequencing reads are available from the National Center for Biotechnology Information (NCBI) under the accession number PRJNA1040016. The authors confirm that the datasets supporting the findings of the cases are available from the corresponding author upon request.
